# Beyond Traditional Flexibility: The Jurdan Sprint Table Test for the Evaluation of Pelvis–Femur Coordination

**DOI:** 10.1111/sms.70179

**Published:** 2025-12-11

**Authors:** Andrea Astrella, Pedro Moreno‐Cabañas, Daniel Iordanov, Pedro Jiménez‐Reyes, Jurdan Mendiguchia

**Affiliations:** ^1^ International Doctoral School Rey Juan Carlos University Madrid Spain; ^2^ Department of Muscle Science RX2 Sports & Health Madrid Spain; ^3^ Sports Department Universidad Politecnica de Madrid Madrid Spain; ^4^ Center for Sport Studies Rey Juan Carlos University Madrid Spain; ^5^ Department of Physical Therapy ZENTRUM Rehab and Performance Center Barañain Spain

**Keywords:** anterior pelvic tilt, flexibility, hamstring injury, kinematics, thigh separation

## Abstract

This study aimed to evaluate the reliability and functional relevance of the Jurdan Sprint Table Test (JSTT), a novel tool designed to assess the dynamic interaction between the pelvis and femurs under high‐speed movement conditions. Thirty physically active male participants completed the JSTT, which involved a rapid kicking motion while kinematic data were captured with inertial measurement units. Eighteen participants repeated the protocol after 24–48 h for test–retest reliability analysis. Intraclass correlation coefficients (ICC) ranged from moderate to excellent (ICC = 0.77–0.97, 95% CI 0.50–0.98). Significant correlations (*n* = 30) were found between static anterior pelvic tilt (APT) and contralateral thigh elevation (*r* = 0.408, *p* = 0.025), and between static APT and sagittal pelvic motion (*r* = 0.517, *p* = 0.003), indicating that individuals with greater static APT tended to exhibit higher contralateral thigh elevation and greater sagittal pelvic motion during the test. Moreover, contralateral thigh elevation was negatively correlated with total thigh range of motion (ROM) (*r* = −0.534, *p* = 0.002). These findings indicate that the JSTT appears to be a reliable method for assessing intersegmental coordination and suggest potential applications in sport performance and rehabilitation screening.

## 1 Introduction

Hamstring muscle injuries (HMIs) are the most prevalent injuries in soccer, with their incidence increasing from 12% to 24% in the past two decades in men's professional soccer [1]. In modern soccer, sprinting is recognized as a crucial element in numerous match‐winning actions [2]. However, sprinting also induces high levels of mechanical strain—considered the primary factor leading to tissue failure—and has been consistently identified as the main mechanism behind HMIs [3, 4]. Accordingly, sprinting should be regarded as the central reference for evaluation, prevention, and rehabilitation strategies addressing HMI risk factors, as it represents the specific task in which these injuries most frequently occur [3, 4]. Among these, flexibility remains a variable of interest for both researchers and clinicians. Greater deficits in hamstring range of motion—particularly as measured with the active knee extension (AKE) test—have been used as surrogate indicators of injury severity, the number of days missed before return to play, and the effectiveness of recovery processes aimed at reducing reinjury risk [5, 6, 7]. Nevertheless, while hamstring flexibility is one of the most studied potential risk factors for muscle injuries, epidemiological studies investigating its effects on hamstring injury and re‐injury rates have produced contradictory findings [8].

These mixed findings might be influenced by how flexibility is traditionally measured. Traditional approaches often isolate flexibility as independent risk factors [8, 9], focusing on static or isolated muscle group assessments without considering broader musculoskeletal and segment interactions. For example, passive hip extension measured with the Thomas test has been shown not to correlate with the actual hip extension observed during running [10], underscoring the limited specificity of these assessments to sprinting mechanics. Such differences may not adequately reflect the neuromuscular demands and strain patterns encountered in real‐world high‐speed tasks. Peak lengths and lengthening velocities may play a critical role in muscle behavior and injury mechanisms, highlighting a significant gap in current clinical assessments. In summary, many traditional flexibility assessments lack specificity to sprinting, particularly in terms of segmental coordination and movement velocity, which should be the reference movement guiding clinical flexibility evaluation. Historically, both strength and flexibility have been studied analytically [8, 9, 11] (e.g., via isokinetic testing, Nordic hamstring exercises, AKE tests), focusing on the isolated contribution of individual segments rather than the complex interrelationships and coordination patterns that drive whole‐body movement.

While these conventional methods provide useful baseline data, they don't capture the complex interplay between muscle groups and joints that occurs during functional tasks, such as sprinting. Conventional flexibility tests [12], such as the Passive and Active Straight Leg Raise [13], Passive and AKE [14], and Askling Test [15], are typically performed by fixing (stabilizing) other body segments (e.g., contralateral femur, trunk) to measure hamstring extensibility in isolation. However, this reductionist approach neglects the segmental coordination and neuromechanical demands, understood as the combined requirements of mechanical loading on the hamstring complex and the motor control needed to manage such loads during sprinting, that characterize actual injury scenarios. In sports activities such as sprinting, flexibility is not merely about the extensibility of a single muscle but involves coordinated interactions between multiple muscle groups and joints (segments) that dictate the final lengthening output of a specific muscle. Specifically, during high‐speed running, modeling studies [16, 17] suggest a quasi‐simultaneous sequence where the maximum stretch of the contralateral iliopsoas precedes anterior pelvic tilt (APT), resulting in a significant proximal lengthening of the ipsilateral biceps femoris. This two‐leg interaction (“switching”), with the pelvis acting as an anatomical lever [18], regulates both strain and energy facilitating interlimb coordination. These findings highlight the need for assessment tools that evaluate dynamic interactions, moving beyond reductionist approaches to better understand real‐world movement patterns and injury risks. Recently, the Jurdan Test [19] was proposed as a tool to assess the dynamic interaction between contralateral hip flexors and ipsilateral hamstrings during movement. While it provides valuable insight into the functional relationship between these muscle groups, its static nature limits its ability to reproduce the velocity and segmental coordination sequence found in sprinting. Evaluating these interactions in a precise and systematic manner is essential to identify deficiencies that static tests may overlook.

The aim of this study is to introduce the Jurdan Sprint Table Test (JSTT) as an innovative tool designed to evaluate the dynamic interaction between the pelvis and femurs during high‐speed movements. (i) The primary goal is to provide an explanation and establish the reliability of the JSTT and its potential to assess key biomechanical variables such as segment orientations. (ii) Additionally, the study explores the internal relationship between key biomechanical variables to provide useful complementary information to common clinical evaluations prior to return to full training and competition. Accordingly, we hypothesized that individuals with greater static APT would tend to exhibit greater contralateral thigh elevation during high‐speed movements executed in JSTT.

## 2 Methods

### 2.1 Participants

A total of 30 male physically active individuals (mean ± SD, age: 25.77 ± 5.32 years; height: 180.57 ± 5.59 cm; body mass: 76.30 ± 3.62 kg) were recruited for this study. According to a recently proposed [20] participant classification framework, the sample predominantly corresponds to the Trained/Developmental level. All participants were physically active, engaged in structured sports practice (e.g., soccer, basketball, athletics) 7.4 ± 1.6 h per week (range 4.7–10.9). Subjects presented no current musculoskeletal conditions at the time of testing and were not specifically excluded if they had a history of an acute hamstring muscle strain‐type injury. Each participant provided written informed consent prior to participation. The study was approved by the Ethics Committee of Rey Juan Carlos University (Approval Number: 0607202217622) and was conducted in accordance with the Declaration of Helsinki. Of the 30 participants enrolled, 18 (mean ± SD, age: 26.72 ± 5.64 years; height: 179.89 ± 4.60 cm; body mass: 76.1 ± 6.0 kg) were included in the test–retest reliability analysis because they were the ones available to perform the second session within the required 24–48 h interval. This restriction was due to the protocol requirement of maintaining 48 h of rest prior to both testing sessions, which limited the feasibility of retesting for some participants. The remaining 12 participants could not comply with this time window due to scheduling conflicts, but all were included in the analysis of internal correlations between the biomechanical variables measured during the JSTT cross‐sectional correlation analyses.

### 2.2 Experimental Design and Procedures

**FIGURE 1:**
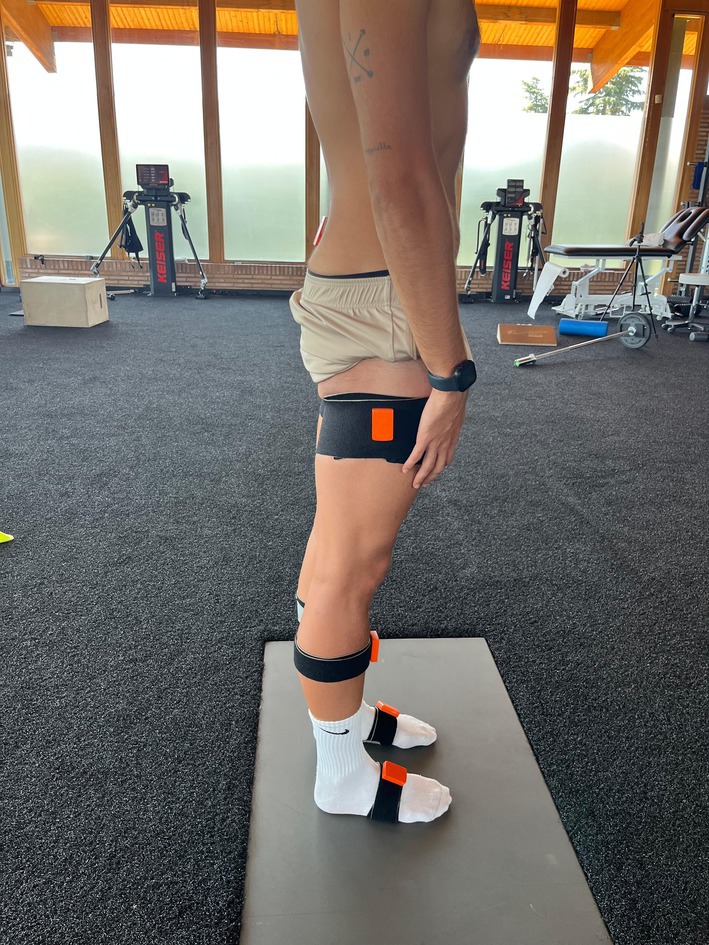
Sensor placement and N‐pose calibration. Wireless inertial measurement units (IMUs) were attached to the pelvis, both femurs, both tibias, and both feet using elastic straps. The pelvic sensor was positioned at the midpoint between the posterior superior iliac spines. The image also illustrates the N‐pose calibration posture, with the participant standing upright on the testing platform, feet shoulder‐width apart, arms relaxed alongside the body.

Kinematic data were collected using seven wireless inertial measurement units (IMUs) (MTw Awinda, Xsens Technologies B.V., Enschede, the Netherlands), sampled at 100 Hz. This system has previously demonstrated valid angle estimates for sagittal plane kinematics in both clinical and sports‐specific field settings [21, 22, 23]. The sensors were placed on the pelvis, both femurs, both tibias, and both feet (Figure 1). The pelvic IMU was directly attached to the skin at the midpoint between the posterior superior iliac spines using double‐sided tape and further secured with overlapping tape.

To determine static APT, participants maintained a relaxed standing position with arms crossed over the chest while a two‐second recording was captured. The pitch value (sagittal plane orientation) derived from the pelvic IMU during this interval was used to quantify APT.

Prior to testing the JSTT, a static pose calibration in the N‐pose position (neutral standing posture with shoulder‐width feet apart and arms relaxed by the sides) was performed for each participant (Figure 1).

**FIGURE 2:**
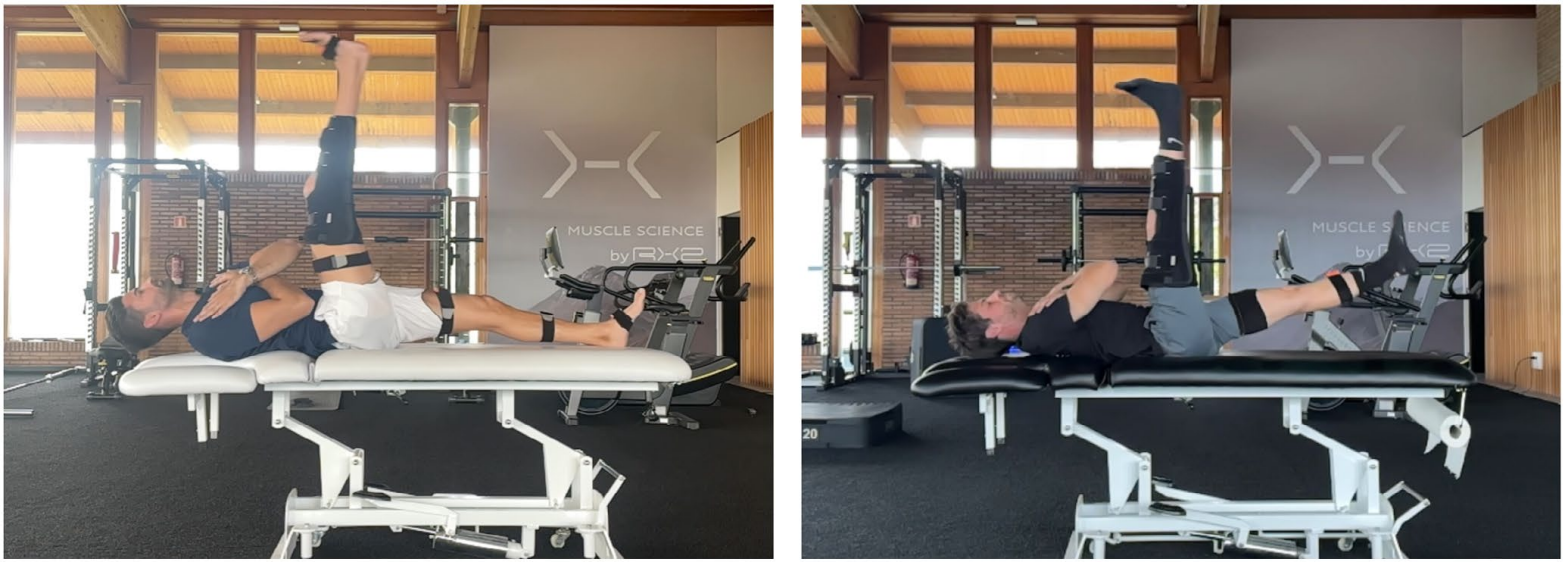
Execution of the Jurdan Sprint Table Test (JSTT). Participants performed a high‐velocity kicking action with the dominant thigh while keeping arms crossed and the non‐dominant leg free to move. The dominant leg was kept in full extension with a knee brace. Sagittal plane segmental orientation was recorded using inertial sensors.

To evaluate test–retest reliability, each participant completed two JSTT sessions separated by 24–48 h. All trials were performed using the participant's dominant leg, defined as the preferred kicking leg, which was secured with a rigid knee brace to ensure full knee extension throughout the movement. To simplify the protocol and reduce testing burden, only the dominant limb was assessed. Participants maintained their arms crossed at shoulder height and were instructed to perform a fast, high swing of the dominant leg with the verbal cue “kick as high and as fast as you can” (Figure 2). No instructions were provided regarding the contralateral leg, as its spontaneous movement relative to the kicking limb was one of the main outcomes of the JSTT. Each session included three maximal repetitions, preceded by one submaximal familiarization trial. Testing conditions, including time of day, setting, and procedures, were kept consistent across both sessions.

### 2.3 Data Analysis

All trials were reprocessed using motion analysis software (MVN Analyze 2021.2, Xsens Technologies B.V., Enschede, the Netherlands) with high‐definition reprocessing in a no‐level scenario, as recommended by the manufacturer [24]. All kinematic data were exported for further analysis in Python (v3.10). This processing included filtering, skin artifact removal, and joint estimation based on the MVN fusion engine [24]. For the analysis, the segment orientation of the pelvis, left thigh, and right thigh, recorded at 100 samples per second, was used. All data were filtered with a low‐pass Butterworth filter, starting from the linear velocity in the sagittal plane for the thighs in order to find the beginning of movement.

**FIGURE 3:**
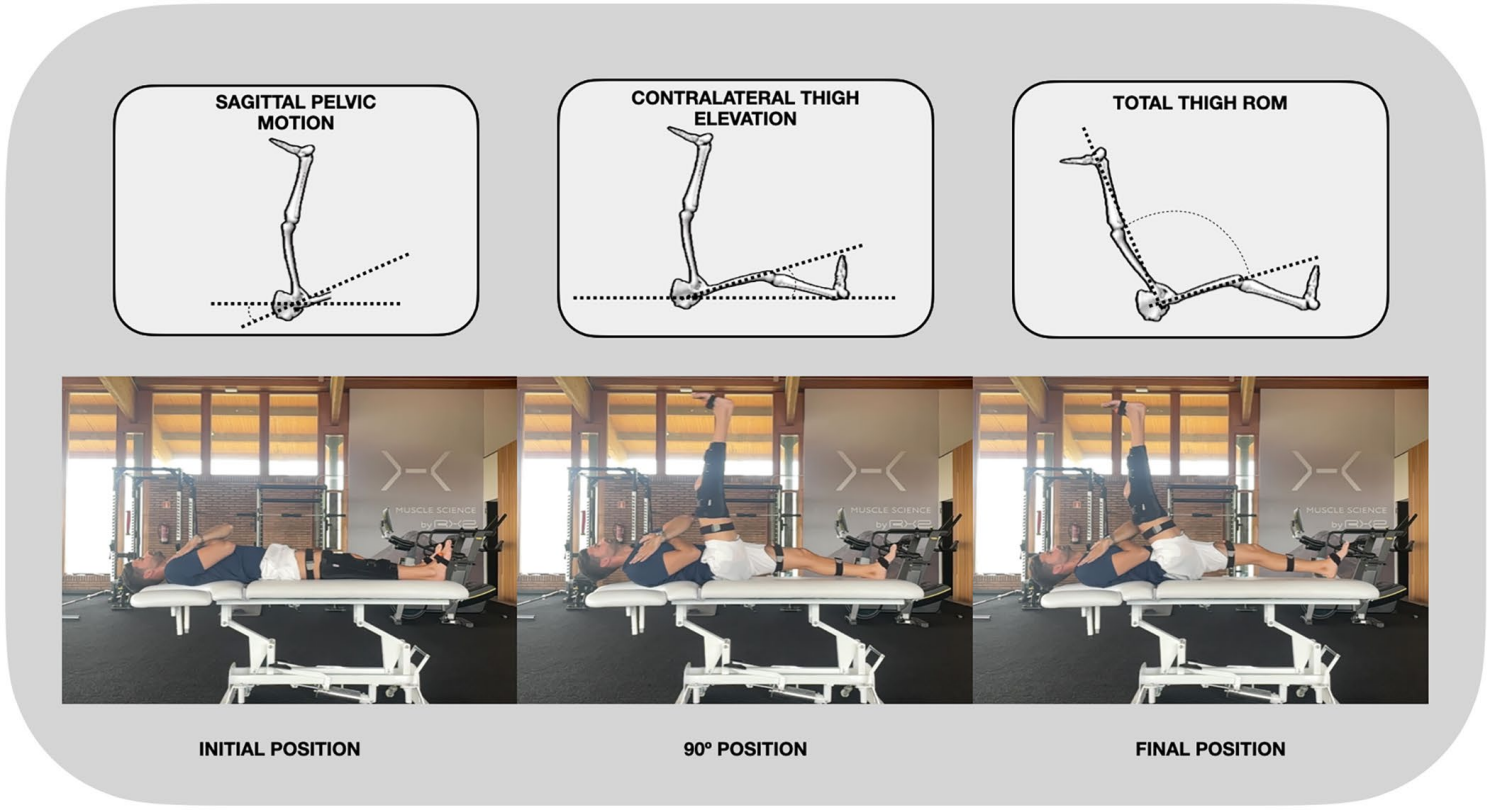
Schematic representation of the three biomechanical variables extracted from the JSTT: Left: Sagittal pelvis motion from the start of movement to when the dominant thigh reaches 90° in the sagittal plane. Center: Total thigh range of motion (ROM) defined as the maximum angle between both femurs during the repetition. Right: Contralateral thigh at the moment the dominant thigh reaches 90° orientation.

To identify the start of each repetition, a velocity threshold of 0.05 m/s was applied. Once dynamic thresholds were established and used to segment the orientation signal, the data from the three repetitions per segment were trimmed accordingly and processed for further analysis. For each repetition, key biomechanical variables were extracted and then averaged across the three trials completed by each participant during each session. These variables included (Figure 3):

1. Total thigh range of motion (ROM) is the maximum angle between the femurs during the entire repetition.
2. Contralateral thigh elevation at the moment the dominant thigh reached 90° of orientation in the sagittal plane.
3. Sagittal pelvic motion from the start of movement to the point when the dominant thigh reached 90°.

For static APT, the pitch value (sagittal plane orientation) derived from the pelvic IMU during the two‐second standing trial was used. The average of this interval was calculated and considered as the representative static APT measurement.

### 2.4 Statistical Analysis

Descriptive statistics are presented as mean ± standard deviation. Statistical analyses were performed using JASP (Version 0.18.2, University of Amsterdam) and Python (v3.10). To assess the test–retest reliability of the biomechanical variables derived from the JSTT (total thigh ROM, contralateral thigh elevation, and sagittal pelvic motion), intraclass correlation coefficients ICC(3,3) with absolute agreement were calculated using a two‐way mixed‐effects ANOVA model (subject × day), following the approach of Shrout and Fleiss [25]. The 95% confidence intervals were estimated through bootstrap resampling with 2000 iterations. For static APT, which was obtained once per session, reliability was assessed with ICC(3,1) For the static APT measurement, which was obtained once per session, reliability was assessed using a two‐way mixed‐effects model, absolute agreement, single‐measure ICC(3,1). All three repetitions from each session were entered into the analysis for every participant, so the ICC reflects the agreement between the sessions derived from these repeated trials. The model assumed that each subject was rated by the same fixed set of raters/tests. The interpretation of reliability was based on the 95% confidence intervals of the ICC [26], rather than the point estimate alone. ICC values were interpreted as follows: < 0.50 = poor, 0.50–0.75 = moderate, 0.75–0.90 = good, and > 0.90 = excellent reliability [27]. Additionally, Typical Error (TE), percentage TE (%TE), and the Minimal Detectable Change at the 95% confidence level (MDC95) were calculated to quantify measurement error in both absolute and relative terms. The TE was obtained as the standard deviation of the differences between test and retest divided by √2. %TE was calculated as: %TE = (TE/|mean of all measurements|) × 100. The MDC95 was calculated using the formula: MDC95 = TE × 1.96 × √2, representing the smallest change that can be interpreted as a real difference beyond measurement error.

Prior to the correlation analysis, all continuous variables were tested for normality using the Shapiro–Wilk test. Since all variable pairs met the assumption of normality (*p* > 0.05), Pearson's correlation coefficient (*r*) was employed using JASP (Version 0.18.2, University of Amsterdam) to assess the linear relationship between them. Correlation strength was interpreted as follows: *r* < 0.30 = weak, *r* = 0.30–0.50 = moderate, *r* = 0.50–0.70 = strong, and *r* > 0.70 = very strong. Statistical significance was set at *p* < 0.05 for all correlation analyses.

Sample‐size estimation for the test–retest reliability component was performed a priori for an average‐measures intraclass correlation coefficient (ICC(3,3)), following established recommendations [28]. Assuming a minimum acceptable reliability of *ρ*_0_ = 0.70, an expected reliability of *ρ*_1_ = 0.85, *α* = 0.05, statistical power of 80%, and *k* = 3 repeated trials per session, the required sample size was estimated at approximately 15–18 participants, which corresponds to the 18 participants included in the present study. For the cross‐sectional correlation analyses, sample‐size adequacy was examined using GPower v3.1. With *α* = 0.05 (two‐tailed) and 80% power, a sample of 30 participants is sufficient to detect a minimum effect size of *r* ≈ 0.46 (Cohen's moderate correlation). Accordingly, while moderate‐to‐strong associations could be identified, smaller associations (*r* < 0.30) may not have been detectable and should be examined in future studies with larger cohorts.

## 3 Results

### 3.1 Reliability

Test–retest reliability results for all JSTT variables, including ICC values with 95% confidence intervals, TE, %TE, and MDC95, are presented in Table 1. Overall, total thigh ROM and contralateral thigh elevation demonstrated excellent reliability, while sagittal pelvic motion showed moderate‐to‐excellent reliability based on the range of the 95% confidence intervals.

**TABLE 1:** Test–retest reliability of biomechanical variables derived from the Jurdan Sprint Table Test (JSTT).

Variable	ICC(3,3)	95% CI	TE (°)	%TE	MDC95 (°)
Total thigh ROM	0.966	0.94–0.98	3.67	3.89	10.17
Contralateral thigh elevation	0.963	0.93–0.98	1.34	15.28	3.72
Sagittal pelvic motion	0.774	0.50–0.90	3.38	24.83	9.37

Abbreviations: %TE, percentage typical error; CI, confidence interval; ICC, intraclass correlation coefficient; MDC95, minimal detectable change at the 95% confidence level; ROM, range of motion; TE, typical error.

The static APT exhibited good‐to‐excellent reliability, with an intraclass correlation coefficient ICC(3,1) of 0.912 and a 95% confidence interval (CI) ranging from 0.780 to 0.966, a TE of 1.34°, %TE of 1.77%, and a MDC95 of 3.18°.

### 3.2 Correlation Analysis

**FIGURE 4:**
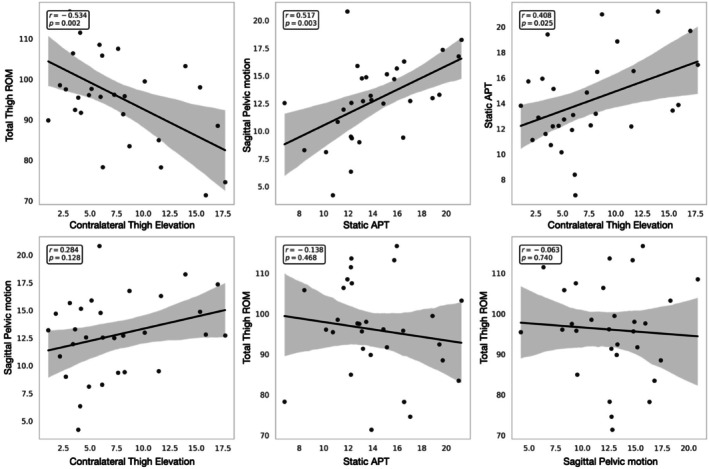
Scatter plots showing the linear relationships among key biomechanical variables extracted from the JSTT. Statistically significant correlations are highlighted with Pearson's *r* and corresponding *p* values. APT, anterior pelvic tilt; ROM, range of motion.

Pearson's correlation analysis (*n* = 30) revealed several statistically significant associations among the biomechanical variables assessed during the JSTT. A moderate positive correlation was found between static APT and contralateral thigh elevation (*r* = 0.408, *p* = 0.025), as well as between static APT and sagittal pelvic motion (*r* = 0.517, *p* = 0.003). A significant negative correlation was observed between contralateral thigh elevation and total thigh ROM (*r* = −0.534, *p* = 0.002). No statistically significant correlations were found between total thigh ROM and either static APT (*r* = −0.138, *p* = 0.468) or sagittal pelvic motion (*r* = −0.063, *p* = 0.740), nor between contralateral thigh elevation and sagittal pelvic motion (*r* = 0.284, *p* = 0.128). A summary of all correlation results is presented in Figure 4.

## 4 Discussion

The present study aimed to assess the reliability and functional relevance of the JSTT, a novel method developed to evaluate the dynamic interaction between the pelvis and femurs under conditions involving high‐velocity limb motion. The findings support both the reproducibility of the test and its value in identifying intersegmental relationships between legs that may be relevant for injury risk. Dynamic variables derived from the JSTT—total thigh ROM and contralateral thigh elevation—showed excellent test–retest reliability. However, sagittal pelvic motion demonstrated moderate‐to‐excellent reliability and relatively higher measurement error, which should be taken into account when interpreting these outcomes in future studies.

Beyond reliability, this study explored the relationship between key kinematic variables to better understand the dynamic behavior of the pelvis and lower limbs during high‐speed movements. A moderate positive correlation was found between static APT and contralateral thigh elevation (*r* = 0.408, *p* = 0.025), as well as between static APT and sagittal pelvic motion (*r* = 0.517, *p* = 0.003), indicating that participants with greater APT in the standing position tended to exhibit higher contralateral thigh elevation and greater sagittal pelvic motion excursion during the test. These findings align with previous research showing that static pelvic posture may influence the dynamic behavior of the pelvis during movement [29, 30]. In fact, previous studies have demonstrated that APT measured in static stance is significantly associated with pelvic kinematics during walking and running [29, 30], reinforcing the functional continuity between static alignment and dynamic control.

**FIGURE 5:**
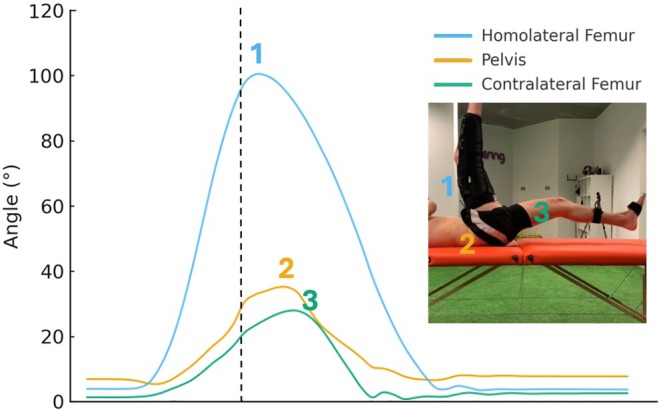
Graphical representation of segmental motion during the JSTT. The figure shows the sagittal orientation (in degrees) of the homolateral femur, pelvis, and contralateral femur throughout the kicking action. The inset illustrates the position of each segment at the moment the dominant thigh reaches 90°.

The contralateral thigh behavior seems to be primarily driven by the preceding movement of the pelvis. During the JSTT, the temporal sequence of segmental motion is reversed compared to sprint mechanics. In sprinting, the maximum stretch of the contralateral iliopsoas precedes APT, resulting in significant proximal lengthening of the ipsilateral biceps femoris [16, 17]. In contrast, during the JSTT, the kicking leg initiates the sequence by first placing initial strain on the hamstrings of that side, followed by a rapid posterior pelvic tilt—opposite to the sequence observed in sprinting—and, ultimately, elevation of the contralateral thigh (Figure 5).

**FIGURE 6:**
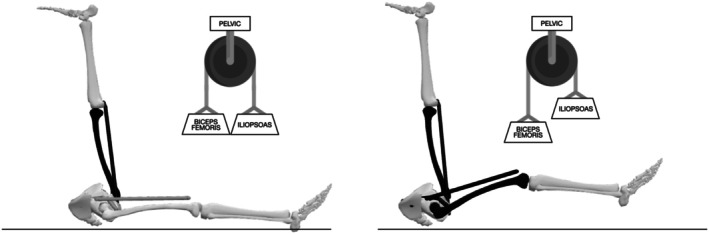
Top panel: Kinematic sequence during sprinting, highlighting the stretch of the contralateral iliopsoas (1), subsequent anterior pelvic tilt (2), and resulting lengthening of the ipsilateral biceps femoris (3). Bottom panel: Segmental coordination during the JSTT. The kicking leg induces biceps femoris stretch (1), followed by posterior pelvic tilt (2), and elevation of the contralateral thigh (3), illustrating the reversed coordination sequence relative to sprinting.

This inverse sequence corroborates that, both during sprinting and in the JSTT, the femurs and pelvis behave as a whole and indivisible system (Figure 6) and therefore should not be analyzed in an isolated and independent manner as has been done up to date. In this context, the JSTT could be regarded as a simplified experimental model that captures relevant aspects of pelvis‐femur coordination rather than a direct reproduction of sprinting dynamics. Concretely, if it is about understanding the underlying structural process that explains the dynamic and natural behavior of lower limbs during high‐speed movements, fixing one of the legs or trunk during testing would not be recommended.

**FIGURE 7:**
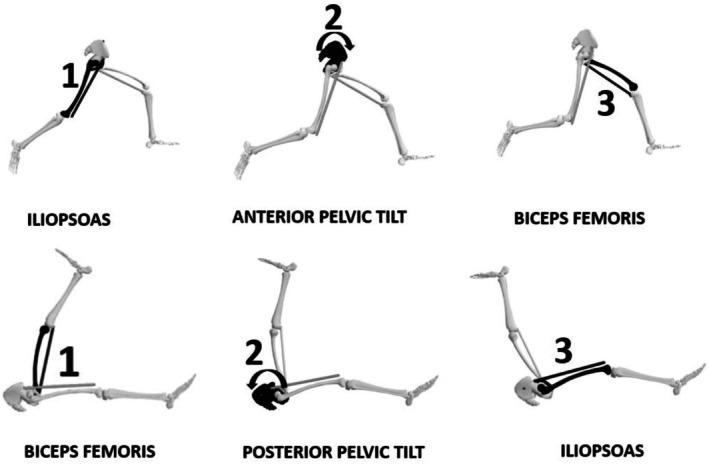
Representation of the pelvis as an anatomical lever modulating the dynamic interaction between the biceps femoris and iliopsoas during high‐speed movement. The schematic illustrates how pelvic motion may redistribute strain and coordinate energy transfer between the posterior (hamstring) and anterior (hip flexor) chains. This mechanism reflects the integrated neuromechanical behavior of the femur–pelvis system during tasks like the JSTT or sprinting, highlighting the pelvis's role as a central structure coordinating bilateral muscle–tendon unit function.

Interestingly, static APT showed a significant positive correlation with sagittal pelvic motion during the test. This may indicate that individuals with a greater APT at rest may adopt a strategy that could also increase pelvic mobility to reduce strain on the kicking leg. However, this same strategy may concurrently increase displacement—and potentially mechanical demand—on the contralateral limb. These findings reinforce the concept of the pelvis functioning as a central anatomical lever (Figure 7) modulating both strain distribution [18, 31] and energy transfer between limbs as previously suggested.

This potential regulatory role of the pelvis appears tightly linked to the coordination between contralateral thigh elevation and total thigh ROM, which showed a moderate negative correlation (*r* = −0.534, *p* = 0.002). In this context, a greater elevation of the contralateral thigh may constrain maximal thigh separation, which could hypothetically reflect a neuromechanical strategy that redistributes tension or adjusts movement through pelvic compensation. These intersegmental dynamics mirror those observed in sprinting. In both the acceleration [32] and the maximum velocity phases [33, 34], thigh separation has been shown as an essential requirement for developing key mechanical capabilities (higher angular velocities, greater ground reaction forces, and shorter contact times) and therefore maximizing performance. Future studies should examine whether the JSTT, in terms of thigh separation and pelvic position, is transferred to these sprint phases. These findings raise the possibility that a simple clinical test performed on a table could provide preliminary insight into the dynamic interaction between the pelvis and femurs. However, whether the JSTT can predict total ROM between the thighs and pelvic position during sprinting remains to be validated. Future studies should therefore examine the associations between JSTT‐derived variables and pelvic/thigh kinematics during high‐speed running to confirm its potential utility as a surrogate for sprint‐specific assessments. If so, it would become a valuable tool in prevention, rehabilitation, and during the return to play, being a surrogate measure of the strain bearable by the femur‐pelvis system and specifically by the hamstring muscles.

The results of the present study reinforce the premise that flexibility and range of motion should not be evaluated in isolation, but rather as part of a broader functional system. Traditional flexibility assessments often fail to account for inter‐limb dynamics or pelvic contributions. The JSTT addresses this gap by capturing bilateral interactions, making it a promising tool for screening and longitudinal monitoring in populations at risk for hamstring injuries and for identifying performance‐related biomechanical markers.

Moreover, variables such as total thigh ROM and contralateral thigh elevation demonstrated excellent reliability, although the latter showed a moderate percentage error, representing a promising marker for future applied research. Its MDC95 ≈ 4° provides a practical threshold, indicating the minimum change that can be considered real beyond measurement error. This benchmark enhances its clinical and applied relevance for longitudinal monitoring could represent promising markers for future applications. This makes them particularly suitable for use in high‐performance sport and rehabilitation environments, where longitudinal monitoring is essential. Future studies should examine the feasibility and validity of tracking these variables through accessible tools (e.g., video‐based analysis) in field conditions to enhance their translational utility in clinical and performance settings.

While the test demonstrated robust reliability and potential practical relevance, the current study was limited to a cross‐sectional design and a relatively homogeneous sample composed exclusively of males, which may limit the generalizability of the findings to female populations. In addition, prior hamstring injury history was not systematically stratified, which precluded subgroup analyses and sensitivity comparisons. Another limitation is that data were sampled at 100 Hz; using higher sampling frequencies could potentially reduce measurement error (TE, %TE, MDC95) and further improve accuracy. Future studies should examine the predictive validity of JSTT variables in prospective cohorts, explore their responsiveness to specific interventions, and validate their associations with sprinting mechanics and hamstring injury risk.

## 5 Perspectives

This study assessed the reliability and functional relevance of the JSTT, demonstrating that several of its biomechanical variables, particularly total thigh ROM and contralateral thigh elevation, show excellent test–retest consistency and practical applicability. Importantly, this study explored the relationship between key kinematic variables to better understand the dynamic behavior of the pelvis and lower limbs during high‐speed movements. The findings support the idea that static pelvic posture is linked to dynamic pelvic function, reinforcing the pelvis's role as a central anatomical lever coordinating interlimb mechanics. These findings suggest that the JSTT is a reliable and feasible method to evaluate dynamic pelvis–femur interactions, with potential applications in sport performance and rehabilitation contexts. Future research should investigate whether JSTT variables predict sprint mechanics and evaluate their responsiveness to training interventions in real‐world environments.

## Funding

The authors have nothing to report.

## Ethics Statement

Approved by the Ethics Committee of Rey Juan Carlos University (Approval Number: 0607202217622).

## Conflicts of Interest

The authors declare no conflicts of interest.

## Data Availability

The data that support the findings of this study are available on request from the corresponding author, A.A. The data are not publicly available due to restrictions (e.g., their containing information that could compromise the privacy of research participants).
